# Stability Studies, Biodegradation Tests, and Mechanical Properties of Sodium Alginate and Gellan Gum Beads Containing Surfactant

**DOI:** 10.3390/polym15112568

**Published:** 2023-06-02

**Authors:** Natalia Stachowiak, Jolanta Kowalonek, Justyna Kozlowska, Aleksandra Burkowska-But

**Affiliations:** 1 Faculty of Chemistry, Nicolaus Copernicus University in Torun, Gagarina St. 7, 87-100 Torun, Poland; jolak@umk.pl (J.K.); justynak@umk.pl (J.K.); 2 Faculty of Biological and Veterinary Sciences, Nicolaus Copernicus University in Torun, Lwowska St. 1, 87-100 Torun, Poland; wodkow@umk.pl

**Keywords:** wet wipes, biodegradation, surfactant, beads, mechanical properties, stability studies, sodium alginate, gellan gum

## Abstract

The excessive presence of single-use plastics is rapidly degrading our natural environment on a global scale due to their inherent resistance to decomposition. Wet wipes used for personal or household purposes contribute significantly to the accumulation of plastic waste. One potential solution to address this problem involves developing eco-friendly materials that possess the ability to degrade naturally while retaining their washing capabilities. For this purpose, the beads from sodium alginate, gellan gum, and a mixture of these natural polymers containing surfactant were produced using the ionotropic gelation method. Stability studies of the beads by observing their appearance and diameter were performed after incubation in solutions of different pH values. The images showed that macroparticles were reduced in size in an acidic medium and swelled in solution of pH-neutral phosphate-buffered saline. Moreover, all the beads first swelled and then degraded in alkaline conditions. The beads based on gellan gum and combining both polymers were the least sensitive to pH changes. The compression tests revealed that the stiffness of all macroparticles decreased with the increasing pH of the solutions in which they were immersed. The studied beads were more rigid in an acidic solution than in alkaline conditions. The biodegradation of macroparticles was assessed using a respirometric method in soil and seawater. It is important to note that the macroparticles degraded more rapidly in soil than in seawater.

## 1. Introduction

Single-use plastics (SUPs) were invented for modern society and are commonly used fast-moving consumer products thrown away after a single use [1]. Plastics include a range of synthetic polymers such as polyethylene (PE), polyvinyl chloride (PVC), polypropylene (PP), polystyrene (PS) or polyurethane (PU), which were applied in most industries [2,3]. Despite various policy initiatives on plastics, the production of plastics in 2020 reached 367 million tons. Cumulative global plastic production is forecast to be 2600 million tons by 2050 without a worldwide ban convention [4,5]. Worn-out disposable products, such as bags, food packaging, protective masks, gloves, and wet wipes, end up in landfills or aquatic reservoirs. Single-use plastic pollution is eroding our ecological environment at an alarming rate worldwide, because plastics are highly resistant to decomposition [6]. The key to stopping plastic pollution is the implementation of biodegradable polymers to develop eco-friendly materials [7]. There are already eco-alternatives for many materials, e.g., biodegradable packaging, disposable dishes, straws, and bags. Furthermore, wet wipes for personal or domestic usage are a significant source of plastic waste. It should be emphasized that replacing non-degradable cleaning wipes is difficult because a surfactant must be introduced. It is also worth noting that commercial wet wipes available on the market are constantly moist, additionally enclosed in a non-degradable package. An approach that can help solve this crisis is the design of biodegradable materials with washing properties due to entrapped surfactants in macroparticles.

Several natural polymers have been widely used as polymer matrices to develop functional beads. The polymer beads generally consist of the polymer matrix and loaded active substance. Sodium alginate (ALG) is an anionic polysaccharide obtained from brown algae, which is composed of two structural units of 1–4 linked α-L-guluronic acid and β-D-mannuronic acid [8]. One of the essential characteristics of alginates is the ability to undergo ionotropic gelation, which is the gel formation process that occurs in contact with divalent cations [9]. Alginates have found application in the food, medical and cosmetic industries due to their beneficial properties such as biocompatibility, biodegradability, and a high capacity to incorporate and release active substances [10,11,12,13]. Wang et al. fabricated sodium alginate/ZnO hydrogel beads containing curcumin to design the delivery systems to entrap and control the release of unstable drugs. The results showed that the composite hydrogel beads protected the curcumin from light degradation and could therefore prolong the antioxidant effect of curcumin [14]. Traffano-Schiffo et al. entrapped lactase in alginate beads in order to maintain its enzymatic activity toward freezing, freezing/thawing, and storage [15]. Moreover, spherical microparticles of sodium alginate and a mixture of sodium alginate and starch can be used as abrasive ingredients in peeling formulations and successfully replace commercial synthetic particles in cosmetics [16].

Another natural polymer used to prepare beads is gellan gum. Gellan gum (GG) is a bacterial exopolysaccharide that contains repeating units of β-D-glucose, L-rhamnose, and D-glucuronic acid [17]. Gellan gum is applied as a suspending, stabilizing, and thickening agent, and its valuable features are stability to heat and changes in pH [18]. This polymer also forms gel beads simply by combining a coil–helix transition and ionotropic gelation with cations [19]. Beads based on gellan gum were applied mainly in active substance delivery systems. Prezotti et al. prepared gellan gum grafted pectin beads using trivalent cation as a crosslinker and ketoprofen as a model drug. They observed that all beads presented high muco-adhesiveness and that their swelling and erosion behavior strongly depended on pH [20]. Osmałek et al. evaluated the properties of gellan macroparticles with the potential application as carriers for oral delivery of meloxicam (MLX) in the prophylaxis of colorectal cancer [21].

There are several studies about the combination of alginates and gellan gum. Park et al. produced Pseudomonas-aeruginosa-encapsulated alginate/gellan gum microbeads to investigate the biodegradation of diesel-contaminated groundwater. The results confirmed that encapsulation could protect microorganisms from toxic contaminants, and the activity of microorganisms could be maintained with the protective barrier of biodegradable molecules [22]. The combination of gellan gum and alginates is also applied in pharmaceutical and medical fields. Jana et al. fabricated aceclofenac-loaded alginate/gellan gum microspheres using maleic anhydride-induced unsaturated esterification for prolonged aceclofenac release [23]. Shirsath et al. designed and optimized vildagliptin (VLG)-loaded gellan gum/sodium alginate beads for sustained release delivery [24].

The creation of cleaning wipes involves the addition of surfactants to the materials. Alkyl polyglucosides are non-ionic surfactants consisting of a hydrophilic sugar moiety linked to a hydrophobic alkyl chain. They are produced from renewable raw materials, such as fatty alcohols (usually from coconut, palm, or rape-seed oil) and glucose (typically from corn, potato, or wheat starch) [25,26]. Decyl glucoside is a mild surfactant with emulsifying, cleansing, and foaming properties commonly used in cosmetics. This surface-free agent is eco-friendly due to its complete biodegradation [27,28].

Our research aimed to produce biodegradable polymer beads containing surfactant (decyl glucoside), which could be a component of eco-friendly wet wipes. Sodium alginate, gellan gum, and a mixture of both natural polymers were used to prepare macroparticles with an inotropic gelation method and calcium chloride as a crosslinker. Although both biopolymers were used to produce the beads earlier, in this experiment, we used them to entrap the surfactant, which has not been tested before. The obtained beads in their wet form and after immersion in different pH solutions were characterized by compression tests to determine Young’s modulus according to Hertz’s approach. The influence of pH on the structural integrity of the beads were defined by exploring their stiffness after exposure to solutions with diverse pHs. The stability of the samples in solutions of different pHs was examined owing to various applications of the beads with a surfactant in products that come into contact with the skin or in household chemicals. This analysis aimed to assess the effects of pH variations on maintaining the bead integrity and functionality, which are crucial for ensuring their efficacy. The investigation involved subjecting the beads to a pH ranging from acidic to alkaline solutions, simulating scenarios where the beads might encounter different environmental conditions. By monitoring changes in the bead appearance and diameter by optical microscope under varying pH conditions, valuable insights were gained into their behavior and potential applications. Biodegradation in soil and seawater of the macroparticles was determined using a respirometric method by OxiTop apparatus, which measures the rate of oxygen consumption by microorganisms (biological oxygen demand) to indicate their metabolic activity during the degradation process.

## 2. Materials and Methods

### 2.1. Materials

Sodium alginate (ALG) was supplied by BÜCHI Labortechnik AG (Flawil, Switzerland), for which the viscosity average molecular weight was determined in our laboratory, and it was equal to 55,800 for K = 0.0178 cm^3^/g and a = 1 [29]. Gellan gum (GG) was purchased from Sigma-Aldrich (Poznan, Poland). Calcium chloride (CaCl_2_), acetic acid (CH_3_COOH), sodium acetate (CH_3_COONa), disodium phosphate (Na_2_HPO_4_), monosodium phosphate (NaH_2_PO_4_), sodium carbonate (Na_2_CO_3_), and sodium bicarbonate (NaHCO_3_) were supplied by Chempur (Piekary Slaskie, Poland). Decyl glucoside (DG) was acquired from Greenaction (Kielce, Poland). All used chemicals were of analytical grade.

### 2.2. Polymer Bead Preparation

The microspheres were produced from sodium alginate and gellan gum with incorporated surfactant (Lauryl Glucoside) by the inotropic gelation method. First, gellan gum solution (1.5% *w*/*w*), sodium alginate solution (1.5% *w*/*w*), and a mixture of gellan gum (0.75% *w*/*w*) with sodium alginate (0.75% *w*/*w*) were prepared. The surfactant (1% *w*/*w*) was added to all solutions. Deionized water was the solvent of all components. Next, beads were formed by dripping the polymer solutions from a syringe (diameter of 1.2 mm) to 0.5 M CaCl_2_ solution under constant stirring. The macroparticles were kept in a crosslinking solution for 1 h. Then, they were washed 4 times with deionized water. Three types of beads containing washing agents were produced: beads made of sodium alginate (ALG), gellan gum (GG), and sodium alginate/gellan gum (ALG + GG) mixture.

### 2.3. Studies of Macroparticles in Different Conditions

The solutions of different pH were prepared: acetate buffer (pH = 4, pH = 5), phosphate buffer (pH = 6, pH = 7, pH = 8), 1% (*w*/*v*) NaHCO_3_ solution (pH = 9), and 1% (*w*/*v*) Na_2_CO_3_ solution (pH = 10) to study the stability of the prepared macroparticles. The obtained polymer beads were immersed in these solutions for 2, 4, and 24 h. After each time, the appearance of macroparticles was observed by the optical microscope Motic SMZ-171 BLED (Hong Kong, China) at a magnification of ×10. The diameter of the beads was measured using this microscope. The images and sizes of the beads stored in the deionized water were also obtained.

### 2.4. Mechanical Tests of Polymer Beads

Mechanical properties of the sodium alginate and gellan gum particles were conducted at room temperature using a mechanical testing machine equipped with compression jigs (EZ-Test SX Texture Analyzer, Shimadzu, Kyoto, Japan). The beads soaked in solutions of different pH (pH = 4–10) for 2 h were examined. The tests were carried out at a 1 mm/min compression speed. The Hertz theory was used to determine Young’s modulus. Hertz’s model describes the relationship between force and displacement for an elastic sphere compressed between two flat smooth surfaces, according to the following equation:

(1) $$F=\frac{{4R}^{\frac{1}{2}}}{3}E*{\left(\frac{H}{2}\right)}^{\frac{3}{2}},$$

where *F* is the applied force, *R* is the initial radius of the bead, and *H* is the displacement [30]. E* is Hertz’s modulus that is related to Young’s modulus by:

(2) $$E*=\frac{E}{1-v^{2}},$$

where *v* is Poisson’s ratio, assumed to be 0.5 [31].

The results were recorded using the Trapezium X software (version 1.4.5, Shimadzu, Kyoto, Japan). The presented data are the average values calculated from 7 measurements for each type of bead.

### 2.5. Biodegradation Studies of Beads

The prepared bead biodegradation in soil and seawater was determined using a respirometric method with a OxiTop Control OC 110 set (WTW, Xylem Analytics, Weilheim, Germany), which analyzed the microbial respiration activity (oxygen uptake).

The biological oxygen demand (BOD) measurement with OxiTop-Control in seawater was performed according to the supplier’s operating instructions. The seawater was collected from the Baltic Sea near Gdansk. The seawater (164 mL) and each type of polymer bead (10 g) were put in 500 mL glass bottles. Nitrification solution inhibitor NTH 600 (3 drops) and the carrier containing CO_2_ absorber (0.4 g NaOH) were added to the bottles. The samples were incubated at 20 °C for 28 days. Seawater was used as a control sample (endogenous respiration). The respiratory activity of microorganisms was demonstrated in mg O_2_ × dm^−3^ of seawater after 28 days.

In the case of biodegradation of the beads in soil, the measured values were also recorded by the OxiTop-Control system in the pressure p mode. The soil (100 g), polymer macroparticles (10 g) and a carrier with CO^2^ absorber (0.4 g NaOH) were put in 1 dm^3^ glass containers. The specimens were incubated at 20 °C for 28 days. The neat soil was used as a control sample (endogenous respiration). Respiratory microbial activity was expressed as mg O_2_ × kg^−1^ of soil after 28 days. The oxygen consumption measurements in seawater and soil for all samples were recorded every two days.

## 3. Results and Discussion

### 3.1. Images and Size of Beads Immersed in Different pH Solutions

The polymer beads based on gellan gum and sodium alginate were formed by ionic crosslinking. The gelation of gellan gum occurs through an ionic chemical bonding between calcium cations and two carboxylate groups from glucuronic acid units in the gellan gum chains. Hydrogen ions from the gellan gum are exchanged with calcium ions [32,33]. The process of sodium alginate crosslinking occurs through the exchange of sodium cations with calcium ones. While every Na^+^ interacts with only one carboxyl group of the alginate chain, the Ca^2+^ interacts ionically with the carboxyl group of guluronate residues, forming a three-dimensional network usually described by the egg box model [34].

**Figure 1:**
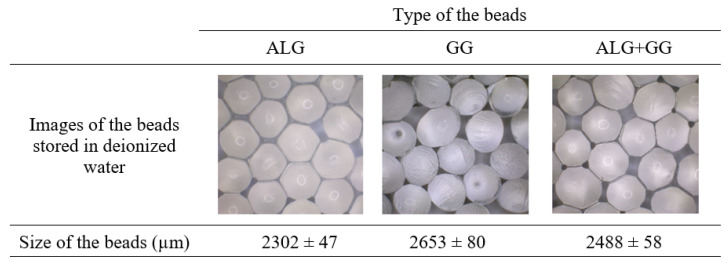
Images and size (diameter) of the wet polymer beads made of sodium alginate (ALG), gellan gum (GG), and sodium alginate/gellan gum (ALG + GG) mixtures.

Figure 1 presents the images and the diameter of the wet beads prepared from sodium alginate, gellan gum, or a combination of both biopolymers. All beads contained surfactant and were stored in deionized water. The polymer macroparticles were spherical. Based on the presented images, the surfaces of the alginate beads were smooth. Along with adding gellan gum, the samples revealed a more ridged surface with tiny cavities. The prepared beads also varied in size. The alginate macroparticles were the smallest (approx. 2300 µm), and the beads made of gellan gum had the largest diameter (approx. 2650 µm). In contrast, the diameter of the beads made of the biopolymer mixture was right between the size of alginate and gellan gum beads, indicating an additive effect. The more compact structure of alginate beads resulted from the presence of guluronate units involved in crosslinking, whereas linear gellan gum created a loser structure. Adrover et al. also prepared gellan gum beads by ionotropic gelation technique. They observed that this method produced a homogeneous population of spherical-shaped beads of similar size [35].

**Figure 2:**
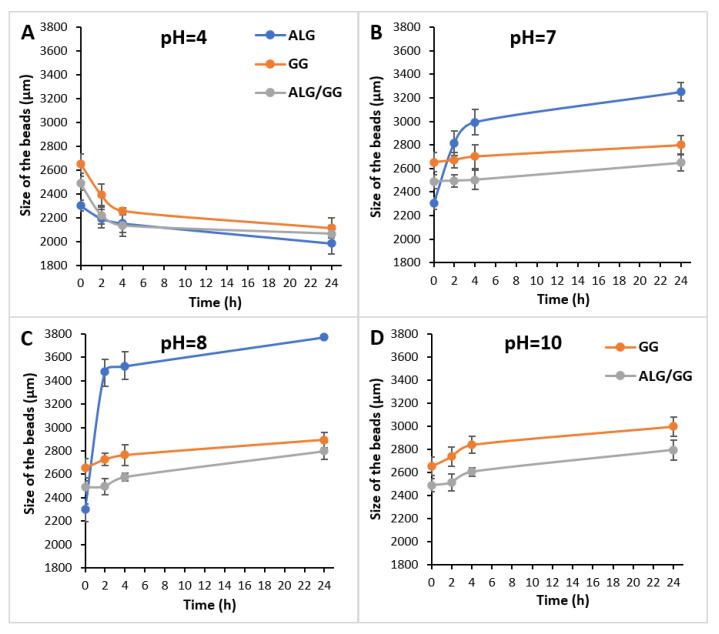
Changes in the size (diameter) of wet beads after immersion for 2, 4, and 24 h in solutions of different pHs: (**A**) pH = 4, (**B**) pH = 7, (**C**) pH = 8, (**D**) pH = 10. At 0 min, the beads stored in distilled water were measured.

**Figure 3:**
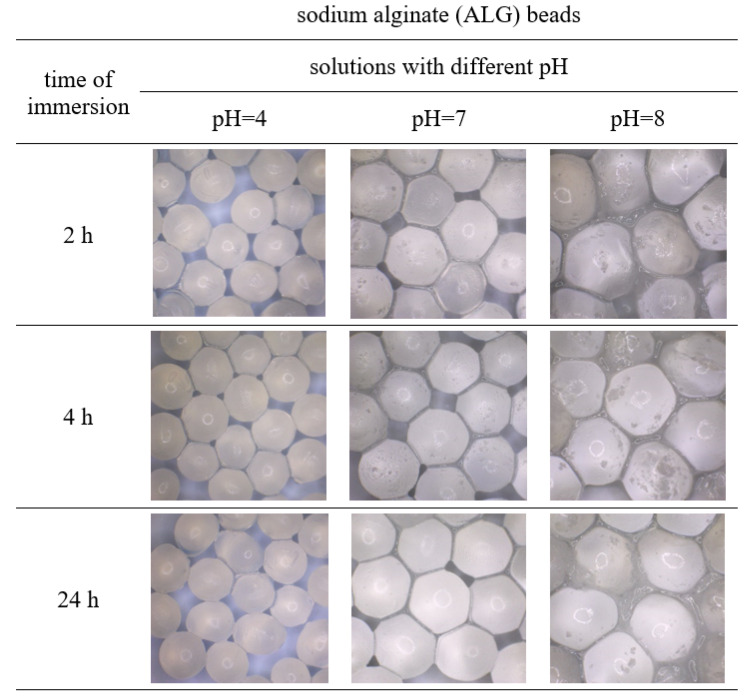
Images of sodium alginate beads (ALG) after immersion in solutions of different pHs for 2, 4, and 24 h.

**Figure 4:**
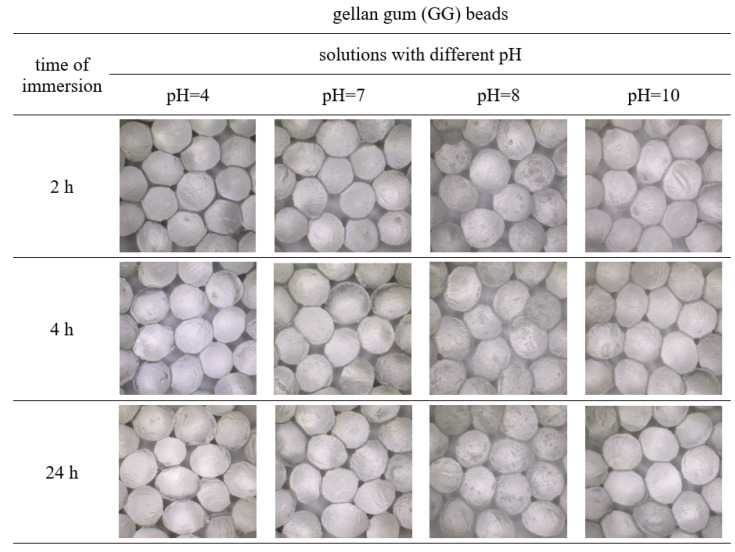
Images of gellan gum beads (GG) after immersion in solutions of different pHs for 2, 4, and 24 h.

**Figure 5:**
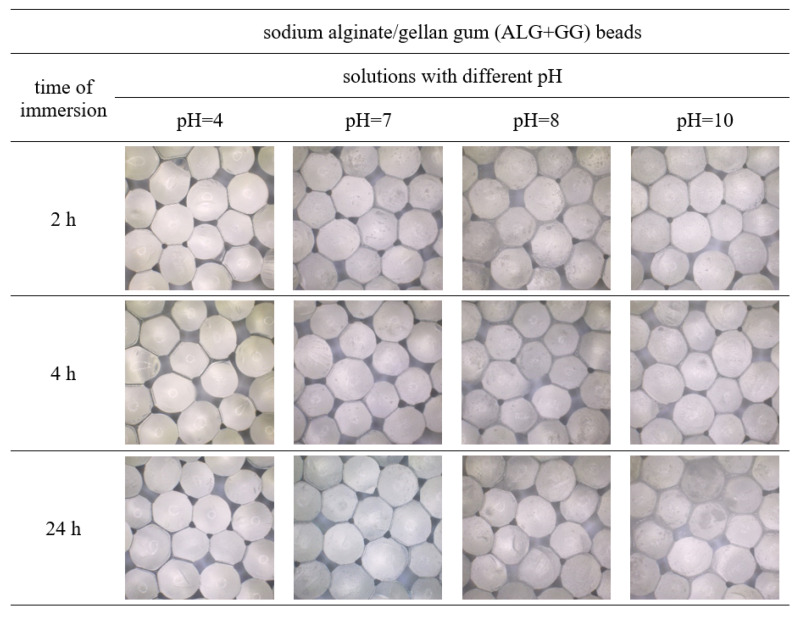
Images of sodium alginate/gellan gum beads (ALG + GG) after immersion in solutions of different pHs for 2, 4, and 24 h.

The obtained polymer beads loaded with the surface active agent were immersed in solutions of different pH for 2, 4, and 24 h. The appearance and diameter of these macroparticles were observed and measured using the optical microscope, which was shown in Figure 2, Figure 3, Figure 4 and Figure 5. Table 1 shows the percentage changes in size of the beads after 24 h of immersion in different pH solutions. The observations were conducted in the pH range of 4–10, but the images of beads immersed in solutions of pH = 4, 7, 8, and 10 were presented because the appearance of the beads after submergence in the solutions of pH = 5, 6, 9 was similar to those illustrated. Thus, only some representative results were shown.

The stability of beads in solutions at different pHs is crucial for their successful application in skincare or personal hygiene products. When these beads come into contact with the skin, it is important that they remain intact and do not degrade too quickly or release their active ingredients when mechanically damaged (after being pressed). These beads can effectively provide their intended benefits and enhance the overall user experience by ensuring stability across a range of pH, from acidic to alkaline.

Similarly, materials used in cleaning agents for domestic use should indicate the appropriate pH values for optimal performance in removing dirt and other contaminants. The pH of a cleaning product can significantly impact its cleaning efficiency. By indicating the appropriate pH range on the packaging, consumers can make informed decisions about the products they choose for different cleaning tasks.

Products containing acidic agents are particularly effective in dissolving limescale and rust. These agents break down the mineral deposits making them easier to remove. Acidic cleaning products are commonly used in bathrooms, kitchens, and other areas prone to the buildup of these substances.

On the other hand, alkaline products are specifically formulated to dissolve fats and grease. Alkaline agents have the ability to break down the chemical bonds in fatty substances, allowing them to be easily washed away. This makes alkaline cleaning products well-suited for tackling greasy kitchen surfaces, oven grime, and other areas where stubborn grease accumulations are common.

In summary, understanding the appropriate pH range for different applications is crucial for developing and using materials, whether for skincare products, cleaning agents, or other purposes. Considering pH compatibility and selecting the right products can ensure optimal performance, effectiveness, and user satisfaction.

The presented images indicate that in the case of beads’ immersion in acidic solution, the macroparticles were reduced in size compared to the initial samples stored in deionized water (Figure 2, Figure 3, Figure 4 and Figure 5, Table 1). The number of hydrogen ions present in a low pH solution made the bead structure tighter and prevented the gel network from destabilizing. This may be related to the osmotic equilibrium by avoiding Ca^2+^ migration into the solution or by strengthening the gel network by diffusing H^+^ ions [36]. Maintaining the integrity of the prepared biopolymers beads in an acidic environment suggests that they can be used in cleaning products for rust and limescale removal. Moreover, the time of immersion in the acidic medium did not affect the stability of all beads significantly. Even after 24 h of incubation, the obtained macroparticles retained their shape and appearance.

It was also noticed that the prepared spheres swelled in phosphate-buffered saline of pH-neutral. The alginate beads showed the highest efficiency of swelling, and this process was more effective in solutions of higher pH (Figure 2B,C, Table 1). The swelling of gellan gum and alginate beads in phosphate buffer (pH 6–8) was associated with the exchange of the crosslinked calcium ions for sodium ions of the dissolution medium [37]. The beads obtained from gellan gum and the mixture of the biopolymers formed a more compact structure than the alginate beads, which resulted in less swelling.

In alkaline solutions, all the samples first swelled and then degraded (Figure 2C,D, Figure 3, Figure 4 and Figure 5, Table 1). Furthermore, the sodium alginate macroparticles were completely disintegrated after 1 h of incubation in the solutions of pH 9 and 10. The most stable beads were obtained from gellan gum and by mixing sodium alginate and gellan gum, making them suitable for cleaning products with an alkaline pH. After 24 h incubation under various conditions, these macroparticles did not change shape and maintained integrity and functionality.

It should be emphasized that swelling supports degradation processes in the case of the prepared polymer beads. Swelling in polymers refers to the absorption of a solvent or liquid, causing an increase in the polymer’s volume [38]. Swelling can lead to degradation by enhanced diffusion, increasing the mobility of reactants or solvents within the polymer matrix. This enhanced diffusion allows for more efficient transport of substances that can induce the disintegration of the materials. As a result, damage to the crosslinked structure of the beads may occur more rapidly. Moreover, the swelling also increases the surface area of the polymer, exposing more polymer chains to the surrounding environment due to the destruction of ionic interactions [39,40,41]. This increased surface area facilitates interactions with degradative agents, leading to accelerated degradation. In addition, the swelling can induce mechanical stress on the polymer structure, causing internal strain or tension. This stress can weaken the polymer chains and make them more susceptible to destruction [42].

### 3.2. Mechanical Properties of Macroparticles

The mechanical testing results of the obtained polymer beads containing surfactant are presented in Table 2. Young’s modulus of the macroparticles after immersion in different pH solutions for 2 h was determined from the force versus displacement slope. It should be mentioned that the prepared beads fractured after compression to 50% of deformation, resulting in the easier release of the loaded surfactant. The polymer network failed, leading to the beads’ irreversible deformation. According to our assumptions, the release of surfactant from polymer beads would primarily occur through a mechanical process involving damaging the beads via compression. By exploring the stiffness of the beads after immersion in solutions of di-verse pH, it was investigated how the pH of the solution influences the structural integrity of the beads.

As one can see in Table 2, gellan gum beads exhibited the highest values of Young’s modulus in the tested solutions of different pH, indicating the stiffest and most durable structure. At the same time, the ALG and ALG/GG samples were more flexible and were characterized by lower Young’s modulus values. The determined elastic moduli values of the beads also depended on the pH of the immersing solutions. The beads’ swelling became more evident when the solution pH increased. During swelling, water molecules enter the beads weakening the interactions between polymer chains. This phenomenon decreased the values of Young’s modulus of all macroparticles when the pH of solutions increased. This process was the fastest and the most efficient for ALG microparticles. The studied beads were more rigid in an acidic solution than in an alkaline medium, in which calcium cations were washed out from the beads owing to the increasing amount of OH^−^ and Na^+^ ions. The beads prepared from the alginate and gellan gum mixture were characterized by the lowest values of elastic modulus after conditioning in pH 4 and 5 solutions, showing the destabilizing effect of mixing both polymers, whereas these beads had higher values of Young’s modulus than alginate beads in the remaining solutions, revealing stabilizing effect of mixing. It is worth noting that the alginate beads disintegrated after 2 h of immersion in the solution of pH = 9.

Chan et al. studied the mechanical behavior of alginate beads and demonstrated Young’s modulus values in the range between 250 and 800 kPa, depending on the bead formulation. Their measurement was performed at a high-speed compression (40 mm/min), and the concentrations of sodium alginate solution used to prepare beads varied from 5 to 50 g/L. It was found that gel beads were considered viscoelastic and could lose liquid under compression [43]. In our study, the elastic modulus value for alginate beads is significantly lower (approx. 56 kPa) in a neutral environment with a 1 mm/min compression speed. The differences may also be due to the presence of surfactant in the beads.

### 3.3. Biodegradation of the Beads

**Figure 6:**
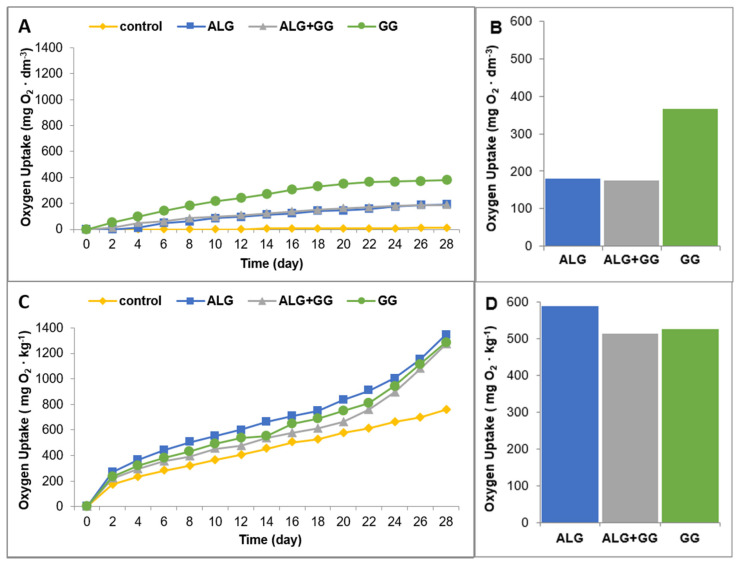
Biochemical oxygen demand in the biodegradation process in seawater (**A**) and soil (**C**) every 2 days in the presence of polymer beads. Total oxygen uptake after 28 days for samples in seawater (**B**) and soil (**D**).

During the aerobic biodegradation process, when O_2_ is readily available, aerobic heterotrophic microorganisms are primarily responsible for the degradation of complex materials, with microbial biomass, CO_2_, and H_2_O as the final products [44,45]. Biological oxygen demand (BOD) related to oxygen uptake depends on microbial respiration activity. The OxiTop respirometry technique relay on air pressure measurement after the absorption of CO_2_ by NaOH pellets [46]. The biopolymers’ degradation results from the action of the enzymes secreted by microorganisms [47]. Oxygen consumption was measured in seawater and soil in the presence of beads based on sodium alginate and gellan gum with the surfactant addition to assess the biodegradability of these macroparticles. Figure 6A–D depicts the oxygen uptake of microorganisms in seawater and soil after the degradation of the prepared samples during the 28 days. Measurements were recorded every two days. By monitoring the oxygen consumption over time, the biodegradation rate of the macroparticles in both soil and seawater can be accurately assessed. This respirometric method provides valuable insights into macroparticles’ environmental fate and helps evaluate their potential impact on ecosystems and develop effective waste management strategies.

The data presented in Figure 6A–D showed that the tested beads degraded at different rates, and it depended on the composition of the beads and the environment in which they were placed. After 28 days of seawater incubation, the highest biological oxygen demand was noted during the degradation of GG beads (365 mg O_2_ × dm^−3^), while oxygen consumption observed for ALG and ALG + GG beads were much lower (about 180 mg O_2_ × dm^−3^). It was found that water microorganisms were characterized by higher metabolic activity (higher oxygen demand) in the presence of ALG macroparticles compared to GG macroparticles.

The biodegradation in the soil of the studies macroparticles occurred differently. The highest oxygen consumption in soil was observed during the biodegradation of ALG beads (590 mg O_2_ × kg^−1^), while oxygen uptake recorded during the decomposition of ALG + GG and GG beads was similar (approx. 520 mg O_2_ × kg^−1^). It should be emphasized that the obtained polymer macroparticles degraded more efficiently in soil than in seawater. Summing up, the beads based on sodium alginate and gellan gum containing washing agent biodegraded during the 28 days.

The biodegradation process of polymers in soil and seawater can differ due to variations in environmental factors and the presence of different biological and chemical agents. Both soil and seawater contain diverse microbial populations that can contribute to polymer degradation. However, the types of microorganisms present and their activity may differ. Soil tends to have a more diverse range of microorganisms, including bacteria, fungi, and archaea, which can enzymatically break down polymers. Seawater typically has a lower microbial diversity, primarily comprising bacteria and marine fungi, which may have specific enzymatic capabilities for polymer degradation in a marine environment [48,49]. In addition, a higher number of microorganisms was found in soil compared to seawater [50,51]. This may explain the phenomenon of faster biodegradation of macroparticles in soil than in seawater.

## 4. Conclusions

The biopolymer beads made of sodium alginate and gellan gum with the addition of a surfactant were obtained using the inotropic gelation technique. Decyl glucoside was used as the washing agent in this experiment. Based on the findings, it is evident that the beads prepared from gellan gum and the mixture of gellan gum and sodium alginate exhibit stability in solutions with pH values ranging from acidic to alkaline. This stability was observed even after 24 h of incubation, as the beads maintained their shape, integrity, and functionality.

Moreover, the stiffness of all macroparticles decreased with the increasing pH of the immersed solutions due to the swelling. The tested samples were more rigid in an acidic than an alkaline medium. All prepared beads loaded with surfactant biodegraded after 28 days of incubation in soil and sea water. This biodegradability is a significant advantage, as it aligns with the global trend of seeking ecological solutions to combat the escalating plastic pollution crisis. The prepared biodegradable beads have the potential to serve as environmentally friendly components in wet wipes, offering both cleaning properties and eco-consciousness. These macroparticles will be suitable for application in products with acidic pH. Moreover, the beads based on gellan gum and a combination of gellan gum and sodium alginate can be utilized in alkaline products thanks to their stability even at pH = 10.

The urgent need to reduce plastic consumption arises from the growing concern over plastic pollution and its accumulation in water environments and landfills. In response to these global challenges, developing ecological solutions such as these biodegradable beads present a promising approach. Incorporating such eco-friendly alternatives into various products can contribute to mitigating the detrimental impacts of plastic waste on the environment.

## Figures and Tables

**Table 1:** The percentage changes in size of the beads (%) after 24 h of immersion in different pH solutions. Values are shown with the standard deviation.

Solutions	Changes in Size of the Beads (%) after 24 h of Immersion in Different pH Solutions		
	ALG	GG	ALG + GG
pH = 4	−13.8 * ± 2.8	−20.3 * ± 0.4	−20.4 * ± 3.8
pH = 7	29.2 ± 1.7	5.5 ± 2.4	6.1 ± 2.4
pH = 8	39.0 ± 1.7	8.3 ± 2.0	11.0 ± 2.2
pH = 10	−	11.5 ± 2.4	11.1 ± 2.7

* minus (−) means a decrease in the size of the beads at pH = 4.

**Table 2:** Young’s modulus of the wet polymer beads with surfactant immersed in different pH solutions for 2 h. Values are presented with the standard deviation.

Solutions	Young’s Modulus (kPa)		
	ALG	GG	ALG + GG
pH = 4	162.6 ± 8.4 ^a^	168.2 ± 9.9 ^a^	122.5 ± 6.9 ^b^
pH = 5	149.9 ± 5.1 ^a^	152.4 ± 7.8 ^a^	113.7 ± 5.8 ^b^
pH = 6	84.1 ± 4.2 ^c^	132.7 ± 8.7 ^a^	105.9 ± 8.6 ^b^
pH = 7	55.9 ± 7.9 ^c^	122.6 ± 7.5 ^a^	96.7 ± 6.8 ^b^
pH = 8	30.1 ± 6.9 ^b^	112.5 ± 10.3 ^a^	91.9 ± 3.4 ^a^
pH = 9	-	105.4 ± 3.4 ^a^	77.9 ± 7.6 ^b^
pH = 10	-	94.8 ± 6.5 ^a^	43.1 ± 4.2 ^b^

One-way ANOVA with Tukey’s post hoc analysis (*p* < 0.05) was performed to compare the results statistically. Different superscripts (^a–c^) within the same row indicate significant differences between the compared values.

## Data Availability

Not applicable.
